# Reply to Nikolaidis, P.T.; Afonso, J. Comment on “Eschweiler et al. Anatomy, Biomechanics, and Loads of the Wrist Joint. *Life* 2022, *12*, 188”

**DOI:** 10.3390/life12081174

**Published:** 2022-08-01

**Authors:** Jörg Eschweiler, Filippo Migliorini

**Affiliations:** Department of Orthopaedics, Trauma and Reconstructive Surgery, RWTH Aachen University Hospital, Pauwelsstraße 30, 52074 Aachen, Germany; migliorini.md@gmail.com

Pantelis Nikolaidis and Jose Afonso published a letter [1] to the editor highlighting some additional aspects that were not addressed in full in our article [2]. The authors mentioned that deeper considerations of (a) the extrinsic muscles of the fingers and (b) the position of elbow and finger joints would have better complemented our work.

First of all, we thank Pantelis Nikolaidis and Jose Afonso for their interest in our work and for their critical and fruitful considerations to improve the understanding of wrist joint biomechanics. In this context, we would like to mention that our article was an initial publication of a Special Issue about wrist joint biomechanics with the intention of giving a brief overview of the state of the art on that topic. Our goal was not to address all aspects of wrist joint biomechanics and all relevant parameters influencing the wrist in one article. This topic is too wide and important for that.

We agree and are aware of the fact that the consideration of the extrinsic muscles of the fingers, and the position of elbow and finger joints influence wrist joint biomechanics. Likewise, the constitution of different muscles in the context of cross-sectional area and neuronal activation is an important point.

Concerning the point “considering all extrinsic finger muscles and their actions on the wrist joint should be discussed in more detail”, we would like to mention that this would be too much for an overview article. It is correct that these muscles also influence the wrist motion and load. The same goes for the forearm posture. The discussion of the influence on wrist strength for the full range of wrist and forearm posture combinations will be enough for a single manuscript. Different articles focus on that topic, e.g., [3,4,5]. They found significant differences in wrist strength that depends on the forearm posture, and they postulate a definite need for further studies. We agree that the maximum range of motion and force production depends on elbow flexion/extension and supination/pronation. Up to date, we have been working on simulation studies in that context. Biomechanical modeling of the wrist and forearm may provide an approach to estimating the relative changes in wrist strength in the context of elbow flexion/extension and supination/pronation.

Concerning the point “including wrist circumduction in the reported flexion and extension (FE), radial- and ulnar-duction (RUD)”, we focused just on FE and RUD because, as you mentioned, the wrist joint has just two degrees of freedom; the main motion is FE and RUD and the circumduction is a combined motion of them. In the case of, e.g., ergonomic investigations, you have to include such motion patterns as circumduction and the dart-throwers motions as often-existing examples of activities of daily living. For a brief overview of wrist biomechanics, we think it is acceptable to focus primarily on FE and RUD.

Pantelis Nikolaidis and Jose Afonso mentioned also that it should be highlighted that all ‘wrist muscles’ and extrinsic finger muscles are multi-articular since they cross over the wrist, and also over the elbow and/or finger joints. Furthermore, their action on the wrist joint as well as the range of motion of this joint depends on the position of the nearby joints, and this will change the moment arms, their degree of shortening or lengthening, and the resulting ability to produce force. We agree with Pantelis Nikolaidis and Jose Afonso: this is an important topic. However, again, for a brief overview of wrist joint anatomy, biomechanics, and loads, there were too many details, and a detailed discussion of these facts would have been better addressed in an extra publication.

We also appreciate the consideration of Pantelis Nikolaidis and Jose Afonso regarding the loads on the wrist joint, and it is noteworthy to mention the variation in these loads and range of motion as a function of the nearby joints. We also agree with this thought. We are just at the beginning of considering all possible parameters that could influence the biomechanics of the wrist joint. This would also, like the other things, be a welcome idea to realize additional investigations about wrist joint biomechanics.

Currently, and also in the past, we are/were working in the field of modeling and simulation of wrist joint biomechanics in the context of therapy planning and patient-specific support [6,7,8,9,10,11,12,13]. Our intention is to completely approximate the wrist via a musculoskeletal biomechanical model, which includes bone, ligaments, muscles, and their geometries and kinematics to investigate surgical interventions and their possible outcomes. Furthermore, the influence of pathologies on muscle-specific force-generating characteristics and the estimation of further parameters influencing the wrist, e.g., muscle moment arms from the wrist joint and their changes, is a milestone of our research [14,15]. The modeling and simulation approach will allow us a systematic evaluation of, e.g., relative muscle strength differences in the context of forearm posture, and via sensitivity analysis in many more posture scenarios than would be feasible in an empirical study. Furthermore, we could evaluate each muscle’s contribution to the joint moment about a particular axis, and how its moment potential is affected by biomechanical factors, such as muscle architecture and orientation [4].

We appreciate the recommendations of Pantelis Nikolaidis and Jose Afonso for further reading of our article by a broad readership, and we will go on to investigate the interactions among the different joints of the forearm (finger, wrist, and elbow joint) in our future works.

## References

[1] Nikolaidis P.T., Afonso J. (2022). Comment on Eschweiler et al. Anatomy, Biomechanics, and Loads of the Wrist Joint. *Life* 2022, *12*, 188. Life.

[2] Eschweiler J., Li J., Quack V., Rath B., Baroncini A., Hildebrand F., Migliorini F. (2022). Anatomy, Biomechanics, and Loads of the Wrist Joint. Life.

[3] La Delfa N.J., Langstaff N.M., Hodder J.N., Potvin J.R. (2015). The interacting effects of forearm rotation and exertion direction on male and female wrist strength. Int. J. Ind. Ergon..

[4] La Delfa N.J., Potvin J.R. (2017). A musculoskeletal model to estimate the relative changes in wrist strength due to interacting wrist and forearm postures. Comput. Methods Biomech. Biomed. Eng..

[5] Plewa K., Potvin J.R., Dickey J.P. (2016). Wrist rotations about one or two axes affect maximum wrist strength. Appl. Ergon..

[6] Stromps J.P., Eschweiler J., Knobe M., Rennekampff H.O., Radermacher K., Pallua N. (2018). Impact of scapholunate dissociation on human wrist kinematics. J. Hand Surg..

[7] Eschweiler J., Allmendinger F., Stromps J.P., Nick H.E., Pallua N., Radermacher K. (2014). Biomechanische Modellierung der Handwurzel. Z. Orthop. Unf..

[8] Eschweiler J., Stromps J.P., Rath B., Pallua N., Radermacher K. (2016). Analysis of wrist bone motion before and after SL-ligament resection. Biomed. Eng./Biomed. Tech..

[9] Eschweiler J., Stromps J.-P., Fischer M., Schick F., Rath B., Pallua N., Radermacher K. (2016). Development of a Biomechanical Model of the Wrist Joint for Patient-Specific Model Guided Surgical Therapy Planning: Part 1. Proceedings of the Institution of Mechanical Engineers, Part H. J. Eng. Med..

[10] Eschweiler J., Li J., Quack V., Rath B., Baroncini A., Hildebrand F., Migliorini F. (2022). Total Wrist Arthroplasty—A Systematic Review of the Outcome, and an Introduction of FreeMove—An Approach to Improve TWA. Life.

[11] Eschweiler J., Stromps J.-P., Fischer M., Schick F., Rath B., Pallua N., Radermacher K. (2016). A Biomechanical Model of the Wrist Joint for Patient-Specific Model Guided Surgical Therapy: Part 2. Proceedings of the Institution of Mechanical Engineers, Part H. J. Eng. Med..

[12] Li J., Nebelung S., Schock J., Rath B., Tingart M., Liu Y., Siroros N., Eschweiler J. (2021). A Novel Combined Level Set Model for Carpus Segmentation from Magnetic Resonance Images with Prior Knowledge aligned in Polar Coordinate System. Comput. Methods Programs Biomed..

[13] Ji C., Li J., Praster M., Rath B., Hildebrand F., Eschweiler J. (2022). Smoothing the Undersampled Carpal Bone Model with Small Volume and Large Curvature: A Feasibility Study. Life.

[14] Eschweiler J., Praster M., Quack V., Li J., Rath B., Hildebrand F., Migliorini F. (2022). Comparison of Optimization Strategies for Musculoskeletal Modeling of the Wrist for Therapy Planning in Case of Total Wrist Arthroplasty. Life.

[15] Eschweiler J., Praster M., Quack V., Michalik R., Hildebrand F., Rath B., Migliorini F. (2022). Musculoskeletal Modeling of the Wrist via a Multi Body Simulation. Life.

